# Sex Work Governance Models: Variations in a Criminalized Context

**DOI:** 10.1007/s13178-020-00452-y

**Published:** 2020-05-22

**Authors:** Genevieve Fuji Johnson, Kerry Porth

**Affiliations:** 1 Department of Political Science, Simon Fraser University, Burnaby, Canada; 2 Vancouver, Canada

**Keywords:** Prostitution policy, Sex work, Criminalization, Ideational frames, Governance models, Canada

## Abstract

**Background:**

Under current laws, sex workers are effectively criminalized, which can lead to harmful impacts beyond arrest and prosecution for sex work–specific offenses, including eviction, search and seizure, surveillance, harassment, and deportation. Although these laws are federal, they are realized in and by policy communities at the municipal level.

**Materials and Methods:**

Based on a qualitative and inductive study of local policy actors affected by or involved in the implementation of prostitution laws, including 65 semistructured interviews in 2014, 2015, and 2016, we identify five different governance models within a shared legal framework of criminalization. We derive these models from an exploration of interactions among actors and organizations based in selected Canadian cities, all of which are bound by federal laws that criminalize the buying of sex thus effectively criminalizing prostitution.

**Results:**

Our study surfaces a diversity of traditional and non-traditional policy players who interpret and implement prostitution laws or advocate for and support sex workers. Focusing on equilibrium moments in relationships among these actors, we identify ideational frames that appear to shape dynamics among them and, in turn, give rise to different governance models.

**Conclusions:**

Our findings of different models within the same, overarching legal context are notable because it demonstrates the variability of a single law when it is implemented in local contexts. This is a contribution not just to understanding how prostitution is governed in particular contexts but also to policy and governance theory more generally. Our findings can serve in future, deductive studies that seek to determine the causes and implications of different governance models in the policy area of prostitution and beyond.

Governance refers to the on-the-ground policy practices by interdependent state and non-state actors working toward policy goals that may or may not be shared (Ansell & Torfing, 2015; Capano, Howlett, & Ramesh, 2015; Colebatch, 2014; Engeli & Allison, 2014; Jessop, 2003; Or & Aranda-Jan, 2017; Stoker, 1998). Governance approaches, in any policy area, emerge from dynamic interactions among local actors and consolidate in terms of the relationships among these actors. Our focus on local relationships is motivated by a long-standing recognition that policy comes to life in a dynamic governance context. Practices, relationships, and communities, as well as ideational frames at play within these communities, are constitutive of policy problems and their solutions (Wagenaar & Cook, 2003). This tendency has implications for laws when implemented in local contexts. In Canada, the policy area of prostitution is especially interesting because, even with reference to a single legal regime, we identify different ways—indeed, different models—of governance in local contexts.

We develop this key insight on the basis of our study of local policy communities in Canadian cities, all of which are bound by federal criminal laws prohibiting the purchasing of sexual services and effectively criminalizing prostitution. Communicating for the purposes of selling sexual services in public places that are or are next to school grounds, playgrounds, or day care centers is also a criminal offense. In addition, advertising the sale of sexual services is criminalized. The term “sexual services” is not defined in the criminal code but is understood to refer to prostitution-related sexual services (https://www.justice.gc.ca/eng/rp-pr/other-autre/c36faq/). Under current laws, sex workers are effectively criminalized as they are implicated in a criminal activity, which can lead to harmful impacts beyond arrest and prosecution for sex work–specific offenses, including eviction, search and seizure, surveillance, harassment, and deportation (http://www.aidslaw.ca/site/the-perils-of-protection/?lang=en).

Although these laws are federal, they are realized in and by policy communities at the municipal level. While federal laws supersede municipal policies, we nonetheless find significant variation in how they are implemented and navigated. We still see significant variation in the relationships among local policy actors and differences in governance models related to these laws.

What appears to account for this variation are ideational frames among actors representing local organizations either affected by or involved in the enforcement of prostitution laws. In this area, we see non-traditional policy actors involved in advocating for sex workers^1^ and providing to them support services; we also see more traditional state actors interpreting and implementing federal laws. These sets of actors can be understood as having ideational frames serving in interpreting situations and representing knowledge (Laws & Rein, 2003). Beyond this epistemic function, frames “serve as a basis for both discussion and action” and provide a guide for “doing and acting” (Laws & Rein, 2003, p. 173). Frames, in other words, help actors to understand policy problems, but they also contribute to motivating them to respond in particular ways. With this in mind, they appear to be crucial in the formation of local governance models.

We center our analysis on policy actors involved in sex work^2^—specifically, prostitution—governance. We examine governance relationships among advocacy and support organizations that are created “by and for” sex workers and organizations that are recognized by sex workers as their allies (i.e., non-traditional policy actors). We also look at the interactions between these “by and for” and ally organizations, on the one hand, and municipal governments and police services (i.e., traditional state actors), on the other. We investigate these policy communities in the largest cities by population in Canada—i.e., Toronto, Montreal, Vancouver, Calgary, Edmonton, Ottawa, and Winnipeg—and identify essential characteristics of ideational frames and corresponding models of governance in moments of equilibria, i.e., in moments when relational dynamics balance and temporarily stabilize.

Our standout finding is that non-state actors involved in governance in these cases share ideational frames concerning the human and labor rights of sex workers, the causes of violence against them, and ways of minimizing this violence. An outcome of this framing convergence appears to be that relational interactions among these actors most closely approximate a model of *collaboration*. The extent to which the features of this human and labor rights frame are not shared by more traditional state actors provides us with an indication as to which other dynamics and models are likely. Where state actors have partially overlapping, adjacent, or oppositional frames in relation to those shared by sex worker rights organizations, they are more likely to be *agonistic*, *siloed*, or *antagonistic*. In some cases, it is too early to tell. In these cases, the governance model is *emergent*. Our findings of different models within the same, overarching legal context is notable because it demonstrates variability of a single law when it is implemented in local contexts and animated by relationships and ideational framing among local actors. This variability may have downstream implications in terms of the capacity of local actors and organizations to address the policy problems associated with prostitution.

We begin with an overview of our methodology. We then identify different ideational frames in play among non-traditional and traditional policy actors involved in sex work governance in each of our cases. We go on to sketch out five governance models. Upon presenting our findings, we conclude by highlighting what may be an important relationship between models of governance and the capacity of that community to meet policy goals. Ultimately, we hope to make a contribution not only to understanding how sex work is governed in local contexts but also to developing governance and policy theory more generally. Our findings can serve in future, deductive studies that seek to determine the cause of different governance models and implications for local policy implementation.

## Methodology

Our study was guided by several basic questions. These include the following: How do different actors and organizations interact in the governance of prostitution in local contexts? Is there variation in these approaches? If so, what are the ways in which the approaches are similar and different? What might account for this variation? What are the implications, if any, of these variations?

Conducting a comprehensive search, we identified the primary non-state organizations in Canada involved in governance activities with a pragmatic emphasis on advocating for and supporting sex workers as opposed to prohibiting prostitution. We did not include in our study prohibitionist organizations—organizations that oppose a harm reduction approach to sex work on the basis of radical, anti-porn/prostitution, and traditional feminist ideology (e.g., Anderson, 2002; Ben-Ishai, 2010; Benoit, Smith, Jansson, Healey, & Magnuson, 2019; Beran, 2012; Bernstein, 1999; Nagle, 1997). These organizations tend to understand prostitution as a manifestation of patriarchal power, which is necessarily coercive and violent (Benedet, 2008; Dworkin, 1992; Farley, 2004, 2006, 2007; Johnston, 2011; Leidholdt, 1993; MacKinnon, 1987; MacKinnon, 2011). Although influential at the federal level in terms of the formulation of the current criminal laws around prostitution (see Johnson, Burns, & Porth, 2017; Shaver, 2019), these organizations are not directly involved in the governance of sex work at the local level. They are therefore not included in this study. The most consequential non-state organizations involved in sex work governance at the local level are those formed by actors who have long been subjected to criminalization themselves, i.e., current sex workers and former sex workers (Oselin & Weitzer, 2013). These “by and for” organizations play critical governance activities, specifically through advocating for the fundamental human and labor rights of sex workers, providing non-judgmental health and housing support to sex workers, facilitating information sharing among sex workers about “bad dates,” and engaging in educational outreach to communities in which sex work takes place. Also included are ally organizations such as frontline charitable organizations, which host drop-in programs, provide referrals, offer legal assistance, and/or offer harm-reduction supports. Ally organizations also include those that provide legal services to sex workers, information to sex workers and allies, and/or campaign for decriminalization. Some community health centers, operating in many respects independently of the state and functioning in ways similar to allies of the sex worker community, were also included in this study. State actors included in the study were municipal governments and local police departments that have either specific programs, policies, or guidelines related to prostitution. We chose Calgary, Edmonton, Montreal, Ottawa, Toronto, Vancouver, and Winnipeg because they are the largest cities in Canada and because they have a sizeable policy community of either “by and for” or ally organizations and municipal governments and police departments focusing on sex work governance. Table 1 lists the number of interviews in each city with actors from “by and for” and ally organizations, municipal governments, and police services.

**Table 1:** Numbers of interviews with actors representing organizations involved in sex work governance

City	“By and for”	Charitable organizations-ally	Community health centers-ally	Municipal governments	Police departments
Calgary	0	2	0	1	0
Edmonton	2	2	0	1	2
Montreal	2	2	0	1	1
Ottawa	1	3	1	1	1
Toronto	4	6	1	1	2
Vancouver	3	6	0	2	3
Winnipeg	0	3	1	1	0

We conducted interviews with one national sex worker rights organization. At the time of writing, certain cities did not have health centers, that we know of, providing sex work–specific services and programs. Also at the time of writing, Calgary and Winnipeg did not have publicly visible “by and for” sex worker organization. The police services in Calgary and Winnipeg were not available for interviews. In addition to interviewing actors representing organizations, we also interviewed a number of independent actors in each city

We engaged in an inductive thematic analysis of documents from and interviews with these organizations. The collection and organization of these materials took place during the fall of 2014, the spring of 2015, and the summer of 2016 (and follow-up correspondence with selective interviewees took place in the fall of 2019) and involved searches for key documents produced by non-state organizations and semi-structured, dialogical interviews with their representatives. Also collected were key documents concerning prostitution-related initiatives by state actors, including municipal governments and police services. Where possible, we conducted semi-structured interviews with these state actors. While we were guided by several basic questions, we were not looking in particular for ideational frames or governance models per se*.* Instead, working inductively to analyze our materials, we identified themes related to how actors frame problems and solutions, the kinds of work they do together (or not, as the case may be), and relational dynamics among them. From this, we were able to sketch different governance models. This research project was guided by an ethics protocol centering on informed and on-going consent, which received ethics approval from the lead author’s university.

### Frames and Models

Based on our analysis, we identify several ideational frames at play in interactions among members of local policy communities. These frames can be broadly understood to include views on problems associated with prostitution and solutions to these problems. These frames appear to give rise to specific relational dynamics, which can be understood in terms of governance models. In this section of our paper, we begin by outlining these frames and present the corresponding models. In the ensuing sub-sections, we delve deeper into each case to substantiate these frames and models.

A key frame espoused by sex worker organizations and their allies centers on the *human and labor rights* of sex workers. The main features of this frame include a recognition of the dignity and agency of sex workers. The primary problem associated with prostitution is its criminalization. Criminalization is seen as the main contributor to violence and stigma against sex workers. By removing criminal laws around prostitution, the problems of violence and stigma against sex workers will be minimized. To achieve the goal of decriminalization, this frame emphasizes the need for a sex worker–centered approach. Once criminal laws around prostitution have been removed, prostitution can begin to be normalized as a service profession regulated by common labor standards. In this way, this frame seeks to advance the human and labor rights of those engaged in sex work.

An adjacent frame, held by certain traditional policy actors, is one that conceptually separates sex work from *human trafficking and youth sexual exploitation*. Human trafficking involves recruiting, transporting, transferring, receiving, holding, concealing, harboring, or exercising control, direction or influence over that person, for the purpose of exploitation, generally for sexual exploitation or forced labor (see https://www.publicsafety.gc.ca/cnt/rsrcs/pblctns/2019-ntnl-strtgy-hmnn-trffc/index-en.aspx#a06). Youth sexual exploitation occurs when an adult engages in sexual activity with a child or youth in exchange for money, drugs, necessities of life, or any other items or coerces children/youth into child pornography (see https://www2.gov.bc.ca/gov/content/safety/crime-prevention/community-crime-prevention/exploitation). This frame results in what can be described as a selective law enforcement focus with a priority on youth involved in prostitution and trafficking victims. This frame does not necessarily collide with the human and labor rights frame, although it has the potential of doing so. In important ways, it runs parallel to the human and labor rights frame in that sex worker rights organizations want also to end trafficking and exploitation. However, sex workers report that this enforcement approach, despite being selective, has adverse consequences for them. It is important to point out that when law enforcement claims to target “youth sexual exploitation,” they may in fact be targeting sex workers; the same is true for “human trafficking” initiatives in numerous provinces across the country (see Butterfly Asian and Migrant Sex Worker Support Network, 2018; Canadian HIV/AIDS Legal Network, 2019; SWAN Vancouver, 2015). In critical ways, when realized in action, this frame can have adverse consequences for sex workers, which sex workers are reporting. Thus, in our study, we view this frame as adjacent but also potentially adversarial vis-à-vis sex workers and their allies.

A frame that necessarily collides with the human and labor rights frame is held by other traditional policy actors in this area. This frame necessarily collides with a rights-based or worker-centered frame in that women involved in prostitution are seen as having no choice or agency. This opposing frame conceptualizes prostitution as *inherently coercive and violent*. From this perspective, prostitution is substantively no different from sex trafficking and sexual exploitation. Proponents of this frame seek to prohibit prostitution as a solution to the coercion and violence it necessarily causes. In terms of governance at the local level, this frame takes shape as proactive law enforcement. Table 2 outlines the frames at play in sex work governance.

**Table 2:** Frames

Frame	Human and labor rights of sex workers	Sex trafficking/youth sexual exploitation vs. sex work	Prostitution as coercion and violence
Problems	Violence exacerbated by criminalization of prostitution and stigmatization of sex workers	Violence exacerbated by some criminal laws; Youth sexual exploitation and sex trafficking	Violence against women and girls by men; Prostitution conflated with sexual exploitation and trafficking
Solutions	Removal of criminal laws around prostitution; Application of labor standards to sex work, provision of harm reduction services, including health care and affordable housing; Realization of human rights of sex workers	Selective enforcement of criminal laws related to youth sexual exploitation and sex trafficking	Criminalization of prostitution; Proactive law enforcement

Surveying relational practices among local policy organizations and communities, we identify equilibrium moments in which the status of ideational frames (i.e., whether they are *shared*, *partially overlapping*, *adjacent*, or *oppositional*) are stable. Focusing on these moments of stability, we then identify essential characteristics of the governance dynamics among them. These characteristics can be organized into five sex work governance models: collaborative, agonistic, siloed, antagonistic, and emergent (Table 3). In the following sub-sections, we sketch in more detail the ideational frames in play, and more fully develop the specifics of each model.

**Table 3:** Frame status and governance models

	Models				
	Collaborative	Agonistic	Siloed	Antagonistic	Emergent
Frame status	Shared	Partially overlapping	Adjacent	Oppositional	Partially overlapping or Adjacent

### Collaborative Governance

A dominant theme in the documents and interviews analyzed for this study relates to practices of agreement-oriented deliberation, as well as interdependence among “by and for” organizations and ally organizations. We see in these practices dynamics of collaborative governance. In the governance literature, collaboration refers to the development and maintenance of a shared understanding and the collective working toward a common set of goals (Keast, Brown, & Mandell, 2007). Participants in collaboration are understood to be interdependent and cooperative in joint action, but they remain autonomous and responsive to their particular communities (Thomson, Perry, & Miller, 2007). Chris Ansell and Alison Gash define collaborative governance as “a governing arrangement where one or more public agencies directly engage non-state stakeholders in a collective decision-making process that is formal, consensus-oriented, and deliberative and that aims to make or implement public policy or manage public programs” (2008, p. 544).

In virtually all of the interviews with actors from “by and for” and ally organizations, we see articulations of collaborative governance. We see themes of lengthy histories, sometimes punctuated by conflicts, but also sustained by values, beliefs, and activities converging on shared understandings of problems and solutions among these organizations. In both the interviews with and documents from “by and for” and ally organizations, there are numerous examples of what can be understood as a shared ideational frame that informs the on-going collaborative practices. We thus see a shared understanding of the critical importance of recognizing the dignity and agency of sex workers, advancing their human and labor rights, advocating for the decriminalization of prostitution, and upholding labor standards in the area.

We see these themes most clearly among “by and for” organizations in Toronto, Montreal, and Vancouver (see Lebovitch and Ferris, 2019). These cities have the highest numbers of well-established sex worker and ally organizations that are collectively mobilized on a daily basis. For example, Sex Professionals of Canada (SPOC), based in Toronto, is perhaps the “by and for” organization best known beyond the communities of sex workers around the world. The Executive Director, Amy Lebovitch, and Legal Coordinator, Valerie Scott, along with Terri Jean Bedford, made history in raising awareness about the harms created by criminal laws around adult prostitution and in successfully challenging the constitutionality of three of Canada’s former criminal provisions concerning prostitution (*Canada* v *Bedford*, 2013). Although six months after this decision, *the Protection of Communities and Exploited Persons Act* was introduced, essentially refashioning the previous criminal laws and thus reproducing the same harms (Johnson et al., 2017; see also Bruckert, 2015; Krüsi, Belak, and Sex Workers United Against Violence, 2018), the contribution of SPOC was very significant. Also in Toronto is Maggie’s, the first sex worker–run education project in Canada, which was founded in 1986 “to assist sex workers in our efforts to live and work with safety and dignity” (Maggie’s, n.d.). Butterfly, an Asian and migrant sex workers support network, also based in Toronto, clearly articulates the shared ideational frame. In their words, Butterfly seeks

to promote safety and dignity for all sex workers, regardless of their gender, race, or immigration status, to enhance access to health, social, labour and legal rights and services, to promote equality and eliminate racism, stigma and discrimination against sex workers in general, and Asian and migrant sex workers specifically, … [and] to advocate for human rights of sex workers and to promote the decriminalization of sex work (Butterfly, 2017).

Similarly, in Montreal, organizations also converge on an ideational frame through which to advance the rights of sex workers by removing criminal laws around prostitution, implementing labor standards in the policy area, and providing harm reduction supports. Stella was established in 1995 and has remained a “by and for” sex workers organization. Stella’s primary mission is to “improve quality of work and life for sex workers…” (Stella, n.d.). In particular, they seek to educate the public about “the different ways that sex work happens” and about the “lived experiences [of] sex workers” (ibid.). Ultimately, they seek for sex workers “the same rights to safety and security that are commonplace for other people” (ibid.). Shortly after the establishment of Stella, the Coalition for the Rights of Sex Workers was formed in Montreal, with a more politically motivated orientation focusing on endeavors to improve working conditions and to decriminalize sex work “as a solution to respecting, protecting, and fulfilling sex workers’ human and labour rights” (Clamen, n.d.). The Coalition no longer exists, yet Stella continues to advocate for legal change on behalf of the broader community of sex worker rights activists across the country.

In Vancouver, there are also multiple “by and for” organizations that collaborate together, including Providing Advocacy, Counseling, and Education (PACE), British Columbia Coalition of Experiential Communities (BCCEC), and Sex Workers United Against Violence (SWUAV), all of which advocate for the removal of criminal laws, call for the upholding of labor standards, and take a harm reduction approach (BCCEC, 2007; PACE, n.d.; SWUAV, n.d.). SWUAV in particular speaks directly to Canada’s history of colonialism and its on-going effect and seeks to address this legacy as expressed through sex work by advocating for an anti-oppression approach to harm reduction, by “fighting against poverty, racism, sexism, transphobia, homophobia and other forms of oppression that contribute to the violence and lack of safety for sex workers” (SWUAV, n.d.). As described by an activist based in Vancouver, “[we’ve] always recognized criminalization as a major contributing factor to the violence that sex workers experience” (Interview 801, 31 October 2014).

Collaboration is a dominant theme in virtually all of the interviews with these organizations. These organizations regularly interact with each other, all understand criminal laws specific to prostitution as greatly increasing risks of violence against sex workers, all conceptualize prostitution as a form of labor, and all engage in harm reduction and empowerment practices for sex workers. Importantly, all seek the decriminalization of prostitution. What we learned from our interviews with these organizations is that they have developed relationships among themselves, a common frame for understanding the problems faced by sex workers, and a shared pragmatic approach to addressing these problems, all of which serves as the foundation of this collaboration. Sometimes there is conflict among them. But, as a former executive director of a Vancouver-based organization states: “I think that over the years, it’s leveled out … and I think that that was for the good for our organizations, because there wasn’t, there wasn’t anything to fight about. There was everything to do together, not to fight about” (Interview 808, 25 November 2014).

There exists much collaboration not only among “by and for” organizations within particular cities but also across the country. This collaboration is seen in the decision in early 2013 to hold a National Day of Action to gain public support for the final appeal of the constitutional challenge to prostitution laws at the Supreme Court. Thirteen organizations, including the Triple-X Workers’ Solidarity Association of BC, PEERS Victoria Society, POWER in Ottawa, Stella, and Maggie’s were involved in planning the event, which was held in seven cities across Canada on June 8, 2013. The event, now focused on bringing attention to the importance of decriminalization, has been repeated in 2014, 2015, and 2016 (NSWP, 2016). Over the past several years, the Canadian Alliance for Sex Work Law Reform has played an important role in bringing many of these groups together. Members of this alliance are “by and for” and ally organizations, all of whom have engaged in collaborative practices, such as appearing as expert witnesses before parliamentary committees, lobbying MPs to work toward decriminalization, and developing other strategies for legal reform (Canadian Alliance, 2014a, 2014b, 2015). As a member puts it, “I guess in the past, it’s been hard to get all women together and cooperating, because we all are very different but … under the Canadian Alliance for Sex Work Law Reform [there’s been] some unity in sex worker organizations coming together” (Interview 811, 21 November 2014). Many sex worker rights organizations in Canada participated in its Montreal meeting in the spring of 2015 and continue regular interactions to strategize toward the goal of removing the existing prostitution laws in Canada and implementing labor standards (Canadian Alliance, n.d.).

In our study, we see that collaboration in local sex work governance takes place among non-state actors and that it may be facilitated by a shared ideational frame advancing the human and labor rights of sex workers. In the following sections, we examine relations between “by and for” organizations and their ally organizations, and municipal governments and police departments. In these cases, we see that ideational frames are not fully shared. Instead, frames are partially overlapping, adjacent, or oppositional. We see that, in terms of relations among non-state and state organizations, none of the jurisdictions studied have dynamics that can be understood as collaborative governance. What appears possible are several other models of governance, including one based on agonism.

### Agonistic Governance

Another dominant theme that emerges from our case materials relates to a distrust among actors that is not necessarily destructive and that can be productive. In particular, we see this dynamic in Vancouver, between “by and for” and ally organizations and the Vancouver Police Department (VPD) and City of Vancouver. We can understand these wary but occasionally productive dynamics in terms of agonism. A defining characteristic of agonistic relations is that, whatever trust may exist, it is not enduring or stable (Johnson, 2015). Agonism can be conceptualized as a form of relational realism in the context of competing interests. As Chantal Mouffe states, power relations and antagonism among competing interests can never really be eradicated and emancipation can never fully be achieved (1999, p. 752). She writes that, “if we accept that relations of power are constitutive of the social, then the main question of democratic politics is not how to eliminate power but how to constitute forms of power that are compatible with democratic values” (1999, p. 752). Democratic politics do not require consensus, deep trust, or a shared conception of the good. Instead, democratic politics seeks to transform antagonism among competing actors into productive moments in which they work together to get things done. In the case of Vancouver, we see a kind of agonistic governance with respect to prostitution (Johnson, 2015).

At the foundations of these agonistic relations appear to be ideational frames that are not fully shared by actors. Problem and solution frames only partially overlap, and relationships periodically either deteriorate or ameliorate. During our period of study, relationships within the local policy community in Vancouver could be understood in terms of an antagonism that over the years yielded to forms of agonism in light of a particularly pressing—indeed, horrific—problem and the critical need to respond to it. The relationships were productive because they were focused on addressing the urgent problem of the serial murder of sex workers with pragmatically acceptable solutions (see McLellan, 2011).

In the 1990s, community organization and mobilization in the city’s Downtown Eastside (DTES) raised awareness about the enormous violence against women generally and sex workers in particular in the neighborhood. All of the interviewees for this study from “by and for” organizations and their allies based in Vancouver, as well as the VPD and the City, articulated views on the importance of pragmatic solutions to reduce this violence against sex workers. Virtually all of the Vancouver-based interviewees noted that this awareness, bolstered by a growing body of evidence focusing on violence being exacerbated by criminal laws and containment policies (e.g., Lowman & Fraser, 1989; Lowman, 2000, 2011), as well as a report after a government inquiry into the murders of 67 women, many of whom were from the DTES and some of whom were engaged in selling or trading sex (see Oppal, 2012), contributed to the development of a partially overlapping solution frame among policy actors in the city of Vancouver. As stated by one member of the enforcement community in Vancouver, “the VPD leans to … a harm reduction approach … for a variety of reasons, the evidence is strongest in support of a harm reduction approach” (Interview 807, 1 December 2014). Finding common ground in an understanding of the problems of violence against and murder of sex workers and an understanding of the importance of harm reduction, these groups of actors were able to focus on specific initiatives.

An important initiative was the VPD’s *Sex Work Enforcement Guidelines* (2013), which are unique in Canada. These guidelines emerged from a long process, which involved collaborative practices among the VPD, Pivot Legal Society, and PACE. Basic principles to guide enforcement were to include ensuring the safety, respect, dignity, and well-being of sex workers and maintaining a proportionality between the risk of a situation and its enforcement response. The *Guidelines* explicitly stated that sex work “involving consenting adults is not an enforcement priority” (VPD, 2013, p. 4). Rather, enforcement was to take place “in situations deemed ‘high risk’ due to the involvement of sexually exploited children/youth, gangs/organized crime, exploitation, sexual abuse, violence, and human trafficking” (ibid., p. 3). They also made clear that “police calls regarding violence against sex workers are a priority for assessment and response” (ibid., p. 5). It is important to point out that, in recent years, the use of these guidelines have been demonstrated to reproduce “harms created by the criminalization of sex workers” (Krüsi, Belak, and Sex Workers United Against Violence, 2018, p. 214).

During this time, the City of Vancouver and “by and for” and ally organizations appeared also to share solution frames. In particular, the City was responsive to the Living in Community project. This project grew out of local concerns in the early 2000s that policies serving to maintain street-based sex work in the DTES were contributing to the vulnerability of sex workers. Motivated by a recognition that change was needed to address issues underlying sex work, “resident groups, business improvement associations, community policing centres, and neighbourhood houses” formed a coalition that included sex workers and advocacy and support organizations (Gibson & Goldstein, 2007, p. 9). In the fall and winter of 2006–2007, Living in Community launched an extensive community consultation process consisting of neighborhood dialogues, focus groups, and an online survey. This process resulted in an action plan of recommendations to make communities healthier and safer through prevention/education, harm reduction/intervention, exiting services, and legal responses (ibid., p. 5). The City would eventually respond by developing plans similar to those recommended by Living in Community, which were passed by the City Council in September 2011 (City of Vancouver, 2011; see also City of Vancouver, 2015).

Vancouver stood out for these agonistically productive relations between non-state organizations and state actors, including the City and local police service. The two sides partially shared an understanding of the most pressing problems associated with prostitution and shared an understanding of harm reduction solutions; however, the shared frames were fleeting during the writing of the *Guidelines*, thus inhibiting the development of a more soundly collaborative model. As put by the executive director of a charitable organization in the DTES, “The women do not trust the VPD whatsoever. … they have a little bit of optimism with the VPD, in that they’re more progressive than any of the other policing departments in Canada … they have a representative that comes to our meetings so we’re being heard, which is great … they’re putting a good face out there, but nothing is really happening” (Interview 811, 21 November 2014).

Based on our study, the most productive relations among non-state and state organizations in our study can be best understood in terms of agonistic dynamics. While limited, these agonistic dynamics nonetheless give rise to occasional but important initiatives in sex work governance in Vancouver. In other jurisdictions, we see less productivity between such non-state and state entities.

### Siloed Governance

In Toronto, we see a model of governance in which non-state organizations that support sex workers engage in a high level of intra-group collaboration but communicate infrequently with the Toronto Police Service and the City of Toronto. Typically, they do not engage with them on possible joint initiatives. We can understand this governance approach in terms of silos. Long a problem in the private sector, siloed managers might share larger organizational goals with outsiders but “strive to work in isolation” (Vandersluis, 2001, p. 11) and “view the opinions of those outside the silo of being of no value” (Capasso & Dagnino, 2014, p. 940). When government is siloed, we see strictly enforced hierarchy, fragmented service delivery, and inconsistent policy (Kennedy, Butt, & Amati, 2016; van Broekhoven, Boons, van Buuren, & Teisman, 2015). In siloed relations, non-state and state organizations are aware of each other but have very little interaction (Aylett, 2013; Termeer, Drimie, Ingram, Pereira, & Whittingham, 2018). There is very little communication between them either to collaborate or to antagonize (Aylett, 2013; Bulkeley, 2010; Termeer et al., 2018). Silos encourage actors to ignore outside influences and focus on a “single problem definition” within an organization, which serves to exclude other organizations (Termeer et al., 2018, p. 91). Siloed actors are not necessarily hostile to “outsiders,” although they certainly can be. With actors “embedded in their own organisational cultures and technical practices” (Aylett, 2013, p. 1386), they develop a “trained incapacity” to perceive – let alone address – issues beyond their purview (Aylett, 2013, p. 1390). This incapacity can effectively create adverse consequences for those who fall beyond their purview, which we see in the case of prostitution governance in Toronto.

We see siloed governance in Toronto. For example, All Saints Church Community Centre, Street Health, Agincourt Community Health Centre, South Riverdale Community Health Centre, the Bad Date Coalition, Sistering, Elizabeth Fry Society–Toronto, Butterfly, Maggie’s, and SPOC share ideational frames concerning the human and labor rights of sex workers. They collectively engage in harm reduction practices and decriminalization advocacy. While a number of these organizations receive funding from government agencies, they carry on with their work independently of state actors, especially the police in recent years.

The Toronto Police Service appear to work within an adjacent ideational frame, which is focused on addressing what they perceive to be crimes involving domestic *human trafficking and youth sexual exploitation*. For example, the Sex Crimes Unit has five separate mandates, including the Sexual Assault Investigative Section, Behavioral Assessment, Child Exploitation Section, Human Trafficking Enforcement Team, and Child and Youth Advocacy Centre. As put by a former police officer in Toronto, “… once we developed [ties with the sex worker community] and established the trust … but then I think the Police Service has now gone strictly in human trafficking as such, but remember they’re very busy. You don’t have [the] resources” (Interview 866, 5 May 2015). Referring to the current priority to end trafficking, as stated by a member of the Toronto policing community, “people don’t realize that it’s their next-door neighbor that’s actually it’s happening to. And it’s young girls. These girls are not choosing to go and do a life of prostitution. They’re being forced. So that’s been our focus” (Interview 860, 1 May 2015).

Similarly, the City also appears to be focusing on domestic human trafficking, with partnerships to provide housing for young women who have been victims of this form of violence and exploitation (Tory, 2015). The City also licenses body-rub parlors and adult entertainment clubs, dancers, managers, and attendants and enforces relevant bylaws, but it has very little communication with “by and for,” service, and ally organizations (Interview 863, 30 April 2015). As put by one individual working for a charitable ally organization, “I often feel like there’s kind of two simultaneous and separate worlds going on and so there’s kind of like the grassroots world of harm reduction and … then there’s like this trafficking wing of things going on at the city which is I find sort of disconnected from like the reality of what’s going on” (Interview 856, 29 April 2015). As another individual states, for the City of Toronto, “the focus seems to be more on the issue of trafficking … the push [is] to address trafficking” (Interview 858, 30 April 2015). The focus of these state actors on different problems and solutions can, in important ways, provide a sphere of autonomy to civil society organizations whose focus is instead on sex workers, their working conditions, and their well-being. A member of the Toronto policing community states

I think that, you know, the Maggie’s and the groups like that are doing – they’re doing it for what they believe is the right reason. And if they help someone from being harmed, that is absolutely fantastic, absolutely fantastic. And if they ever want to work with us on something, we would love that. But, you know, I don’t think abolishing the laws against prostitution is the right idea. We’re not enforcing – the laws aren’t against prostitution. The laws are against exploitation (Interview 860, 1 May 2015).

However, despite these claims, Toronto sex workers and sex worker rights organizations experience adverse consequences of law enforcement in their daily lives. Indeed, law enforcement practices in Ontario are specifically targeting sex work and sex workers under the guise of human trafficking and exploitation (Canadian HIV/AIDS Legal Network, 2019). In the next sub-section, we explore antagonistic dynamics in the context of another city in Ontario, Ottawa, where rather than the adjacent frames of siloed relations, oppositional problem and solution frames are common between state and non-state actors.

### Antagonistic

In Ottawa, relationships between non-state actors supporting or advocating for sex workers and state actors, including the City and police service, can be characterized in antagonistic terms. Antagonistic dynamics are characterized by opposing frames, in this case the frames of human and labor rights and prostitution as sexualized violence. In antagonistic politics, actors simultaneously pursue “their own interests and political projects” while also acting as “the mediators of wider struggles” where they pick winners and losers (Newman, 2014, p. 3299). Relations are adversarial, and “any form of we/they relation … becomes the locus of an antagonism” (Mouffe, 2011, p. 16); they can reify into “paralyzing conflict” (Bond, 2011, p. 170). The conflicting parties tend to make moral evaluations of each other, leading away from seeing each other as respectable political adversaries to a more narrow vision of “the opponent … as an enemy to be destroyed” (Mouffe, 2011, p. 5). Thus, antagonistic politics are characterized by non-state and state organizations operating within overlapping spheres but with opposing objectives. Moreover, there is active opposition and open hostility toward each other. Given the inherent power imbalance, the state seeks to dominate while non-state entities feel under siege.

Both the Ottawa Police Service and City of Ottawa appear to understand prostitution through the lens of sexualized violence—in particular sexualized violence against women—and to be taking a proactive crime prevention approach to the sex trade. This approach necessarily collides with a human and labor rights framing. A member of the Ottawa policing community states that “the very term harm reduction tends to be a lightning rod for creating conflict …” (Interview 837, 6 March 2015). As another put it, “we don’t do harm reduction models … we like to see someone get out of the business instead of maintaining the business in a safe manner. There is no safe manner that we can see” (Interview 834, 18 March 2015).

Since 2012, one of the main priorities of the police service is to address violence against women. With reference to prostitution, this priority is articulated in terms of an anti-trafficking agenda focused on youth, which is similar to that in Toronto. Although the police service claims that they are “not after those women that are involved in the sex trade industry” and that they are “there to help them and support them” (Ottawa Police Service, 2015), prostitution street sweeps are common. As a member of the policing community in Ottawa states, “when it comes to sex trade workers, it’s community complaint driven. … if we’re going to do a prostitution sweep … it would be driven by community need” (Interview 834, 18 March 2015). Responding to community complaints, the service claims that its enforcement priority focuses on the buyers of sexual services and not the sellers.

However, non-state organizations have reports from their participants that they are being arrested during these sweeps for possessing drugs, trespassing, or loitering. As Chris Bruckert and Stacey Hannem have found, “the OPS public commitment to ‘clean up’ the streets of Ottawa and to eliminate the visible signs of disorder culminates in three types of formal legal interaction that move beyond conventional *reactive* law enforcement strategies: targeting ‘known’ prostitutes, overcharging, and boundary restrictions” (2013, p. 301). Several interviewees based in Ottawa claim that, given the OPS’s conflation of sex work with trafficking and their treatment of street prostitution as a public nuisance or a criminal activity to be addressed by street sweeps and local community organizing, the relations with the police are very poor. These dynamics are characterized by not merely a lack of shared but rather oppositional perspectives on problems and solutions and outright antagonism.

### Emergent Dynamics

The last set of dynamics found in this study of sex work governance models differs from the previous in that the relations are too nascent to determine if they are tending toward collaboration or toward agonism. There appears to be goodwill from both non-state and state actors and some productive communication. Antagonism does not appear to be the dominant characteristic of these relationships. While officially, their activities may be siloed, they appear to be moving toward a convergence in problem and solution frames. But it is too early to discern equilibria moments.

In Montreal, relationships between sex worker rights organizations and their allies, on the one hand, and the police and municipal government on the other can be characterized as emergent. As stated by a member of the Montreal police service, “A few years ago, the girl was pursued as like a criminal and now she’s for us like more someone who needs help or, you know, so we are not going to criminalize her …. For us she, if she wants to have help, if she wants to have medical help or whatever, you know we are there for her” (Interview 841, 3 March 2015). However, the officer goes on to note the difficulty with which substantive change is made to long-standing practices: “I know that it’s a long procedure, it’s a hard thing to do to change all the mentality you know? It’s hard, but you know we are doing, we are better and better and better every day because we talk a lot about that. A lot actually” (Interview 841, 3 March 2015).

From the perspective of at least one member of a sex worker rights organization based in Montreal, relationships with the police are patchy, at best. As a commentator states,

In many neighbourhoods over the 20 years we’ve developed very good working relationships with the police where we have people we can call, where we can say, oh, something happened or whatever, and they go, okay, I’ll talk to the officer and all of that. In other neighbourhoods it’s more or less a work in progress. It’s sort of, you know like in Hochelaga, the eastern neighbourhood where it’s one of the best, well we’ve been establishing and nurturing that relationship for 20 years. In another neighbourhood it might be eight to ten years or five years that we’ve been nurturing it. In another neighbourhood, though we’ve been working there for nine or ten years but in-house, I mean in the homes of escorts, this year we started to have a street presence and that brought police attention and community organizational and other attention to us, so … it’s like beginning in Hochelaga 20 years ago, it’s a nascent relationship (Interview 843, 4 March 2015).

Dynamics in Edmonton appear similarly nascent. Non-state organizations appear to have a fairly good relationship with local state entities (Interview 818, 18 February 2015; Interview 820, 18 February 2015). Referring to site visits by members of the Edmonton Police Service, one commentator states that “they kind of look around to see that there are no visible signs of something wrong, no trafficked women hiding, cowering in a room. I mean that’s valid, they have to see that nobody’s underage. … They don’t spend much time; they obviously work hard” (Interview 818, 18 February 2015). As put by a member of the service, “I’d like to engage with our NGOs … but most importantly [with] those in the sex industry, body rub practitioners, those in the back pages, and look at developing our guidelines and philosophies for how we interact with the sex industry” (Interview 824, 27 March 2015). He goes on: “Vancouver, back in 2013, did their guidelines, and that’s a start. That’s educating the police agents, as much as the community, that we’re changing our mindset about the liberties that sex workers have, and to treat them with the dignity they deserve” (Interview 824, 27 March 2015).

Moreover, in 2014, the City of Edmonton established the Taskforce on Body Rub Centres. This taskforce included representation of body rub practitioners, body rub center owners, CEASE, OPTION Sexual Health Association, the Police Service, City officials, and provincial and federal governments (City of Edmonton, 2015). The taskforce engaged in extensive consultations with neighborhood organizations, Catholic Social Services, the Sexual Assault Centre of Edmonton, CEASE, multiple body rub center owners, numerous body rub practitioners, Council of Business Revitalization Zones, and City staff. Its members all agreed on the importance of the safety of sex workers and of communities, and the taskforce produced recommendations for “regulations that reduce barriers to compliance, enhance the health and safety of workers, and are mindful of community concerns” (City of Edmonton, 2015, 11). This includes body rub centers having approved plans for security control and emergency response; practitioners having “information on, and access to, social services and other pathways out of the industry if they so choose”; Alberta Health Services and the City of Edmonton providing practitioners and staff with comprehensive health information; the cost of body rub center licenses being reduced to that of other businesses (e.g., nightclubs and bars); the cost of obtaining a body rub practitioner license being eliminated, or significantly reduced; and a 24/7 translation service being negotiated for “workers who may need information or support in their own language or for City staff who are trying to offer service” (City of Edmonton, 2015, 7–8). The relations between sex workers in Edmonton and the police service and the municipal government appear to be potentially collaborative, with a recent elimination of fees for license renewals, but it is still too early to tell.

## Conclusion

In this paper, we have presented our findings from a qualitative exploration of the governance of prostitution by local actors and organizations in major Canadian cities. Our finding of different models within the same, overarching legal context of criminalization is notable. This is a contribution to a more nuanced understanding of how the same law can vary in terms of implementation and governance from one local jurisdiction to the next. Because prostitution laws in Canada are federal, provincial and municipal laws must accord with them. Given this shared, overarching legal framework, our findings direct attention to less legalistic and more relational dynamics in play within local contexts. Our focus on actors and organizations affected by or involved in the governance of prostitution in local contexts enables us to explore relationships among and between them, which in turn draws us into an exploration of ideational frames that appear to give shape to particular governance approaches. Our study raises theoretical questions about the dynamics within the governance of prostitution, which can be understood as a classic case of morality policy (Wagenaar & Altink, 2012). Where policy governs an area that has historically, and that remains understood, in terms of moral taboo, is it more prone to local variation? Exploring this question is important. As our study highlights, the variations are not simply in terms of degree: Collaboration, agonism, siloes, and antagonism are *fundamentally different.*

Moreover, they have different implications for the governance capacity of local policy communities. Our study suggests insights into what constitutes high, middling, and low capacity from which we can develop hypotheses for future testing. High capacity would appear to exist when there is a collaborative approach among non-state and state organizations to understanding problems and developing responses to them. This kind of collaborative governance exists in none of the jurisdictions covered in our study; however, a high-level of collaboration among non-state actors addressing needs, reducing harms, and seeking to empower sex workers does exist particularly in large urban centers. Collaboration among these organizations in terms of programs, services, and campaigns suggests possibilities for enormous capacity where state actors are willing to engage with sex worker organizations as valuable stakeholders in policy development and implementation, and where productive and sustained relationships can develop over time that involve the centering of sex workers, especially the most marginalized.

Middling capacity appears to characterize jurisdictions where there are agonistic or siloed relations. Where there are agonistic relations, policy communities can cooperate on specific initiatives, which can reduce harms and empower sex workers. Siloed relations can provide the space for sex worker organizations to carry out their programming and campaigning with a reasonable degree of autonomy from the state. Nonetheless, in both cases, relationships between non-state and state organizations are precarious, and siloed relations can easily slip toward antagonistic. As such, the capacity not only to provide services and programs but also to engage in substantive legal reform, to shift public opinion, and reduce stigma is fundamentally limited. Low capacity is likely found in jurisdictions in which there are antagonistic relations that impede the development of stable and effective solutions, based on collective non-state and state action, to problems associated with prostitution.

These conclusions are important because, as we have seen in the complex case of sex work governance, effectively addressing policy problems hinges on the capacity of communities existing at the local level. Our study highlights an unintended consequence of Canada’s prostitution laws, which is that they can be implemented in different ways, which has further consequences for the capacity of local communities and, ultimately, for the coherence of the country’s overarching governance regime for sex work.

## Footnotes

1 We adhere to the definition of sex worker put forward by Peers Victoria. Peers, a sex worker organization based in Victoria, BC, writes that sex worker refers to adults “who exchange sexual services for money which necessarily, but not exclusively, includes direct physical sexual contact with clients” (Peers, 2014). They continue, sex workers include “those who engage in outdoor street-level sex work, as well as those who work indoors in their homes, clients’ homes, or in commercial venues” (Peers, 2014). Stella, a sex worker support and advocacy organization in Montreal, QC, writes that “While sex work refers to a large range of people doing a wide variety of work, it is sometimes important for us to set apart our different experiences to emphasize how intersecting realities position us vis-à-vis the law, clients, working conditions, etc. More specifically in a North American context, the experiences of Indigenous sex workers are important to highlight – Indigenous sex workers are at greater risk of criminalization … [for a range of reasons], including deep-seated racism, discrimination, over-policing, and colonization” (Stella, 2013, p. 2; see also Peers, 2014; for discussions of Indigenous sex workers and decolonial approaches to understanding sex work, see Hunt, 2013; James, 2018; Raguparan, 2018; Sayers, 2018). For discussions of male and Trans sex workers, see Butler Burke, 2018, Fletcher, 2013, and Redwood, 2013.

2 Sex work is a broad term that generally applies to services intended to elicit a sexual response and are provided in exchange for money or other goods. In some cases, this involves direct, physical contact with clients; in other types of sex work, such as “cam work,” there is no physical contact. In the context of this paper, we focus on the governance of the selling of sex involving direct physical contact (Mac & Smith, 2018).
